# Draft Genome Sequence of Broad-Spectrum Antibiotic-Producing Marine *Verrucosispora* sp. Strain FIM060022

**DOI:** 10.1128/MRA.00061-19

**Published:** 2019-04-11

**Authors:** Wei Zhao, Yang Xie, Ru Lin, Zhi-kai Fang, Fei Sun, Hong Jiang

**Affiliations:** a Fujian Provincial Key Laboratory of Screening for Novel Microbial Products, Fujian Institute of Microbiology, Fuzhou, People’s Republic of China; University of Southern California

## Abstract

*Verrucosispora* sp. strain FIM060022 shows multiple biological activities and produces polytype structure compounds, including abyssomicins, proximicin A, lumichrome, denosine, and desferrioxamine-like compounds. We present the draft genome sequence of the strain to help predict the biosynthesis of these compounds, identify further biosynthetic potential, and facilitate directed secondary metabolite production.

## Abstract (precis)

*Verrucosispora* sp. strain FIM060022 shows multiple biological activities and produces polytype structure compounds, including abyssomicins, proximicin A, lumichrome, denosine, and desferrioxamine-like compounds.

## ANNOUNCEMENT

The strain FIM060022 was isolated from a sponge collected from Putian, in the Fujian Province of China, and identified as a *Verrucosispora* sp., which is most closely related to Verrucosispora maris based on 16S rRNA sequencing and morphological and physiological characteristics (GenBank accession number KJ143624) (1–5). Turbidity tests revealed that it exhibited broad-spectrum antibacterial activity against Escherichia coli, Bacillus subtilis, Candida albicans, *Rhodotorula*, Staphylococcus aureus, and Cryptococcus neoformans (3). Its compound diversity also corresponded to the broad-spectrum antibacterial diversity (6). To further probe the relationship between the mechanism of secondary metabolite production and the antibacterial activity, the genomic DNA of strain FIM060022 was extracted using the DNA-winding protocol (7). Whole-genome shotgun sequencing was then performed using the MiSeq sequencing platform (Illumina, Inc., San Diego, CA) after generating paired-end libraries with an insert size of 400 bp (8). The sequences were then filtered using AdapterRemoval version 2.1.7 (9) to remove any 3′-terminal contamination. Using SOAPec version 2.0 software (10), we performed quality correction of all reads based on k-mer frequency, with the k-mer used for correction set to 17. The final genome was assembled *de novo* using A5-miseq version 20150522 (11). All the assembled scaffolds were then subjected to gene prediction using GLIMMER 3.0 (12). The metabolic pathways were explored using the Gene Ontology (GO) and Cluster of Orthologous Groups (COG) databases. The GO annotation of protein-coded genes was performed using the Blast2GO software (13). The annotation of protein-coding genes was completed by the BLAST software, and the database used for BLAST was EggNOG (14). The biosynthesis gene clusters underlying secondary metabolite production in strain FIM060022 were predicted using anti-SMASH (15). A BLASTP search against the NCBI nonredundant protein database was performed for predicting the function of proteins encoded in this gene cluster. Default settings were used for all software.

A total of 2,845,130 clean paired-end reads were generated with a 98-fold depth of coverage of the whole genome. The draft genome sequence of strain FIM060022 contained 29 scaffolds (*N*_50_ value of 453,524 bp) with a total size of 6,426,968 bp and had a mean GC content of 70.93%. The results from GLIMMER 3.0 predicted 5,806 open reading frames (ORFs) with an average length of 978.86 bp and a coding density of 88.43%. Strain FIM060022 also had many predicted protein-coding genes; 3,245 coding DNA sequences (CDSs) were found in 77 functional GO groups, and 3,269 CDSs were found in 21 COG groups. Among these, 178 CDSs were associated with secondary metabolite biosynthesis, transport, and catabolism (3.07% of the total draft genome), 395 CDSs were associated with amino acid transport and metabolism (6.80%), 147 CDSs were associated with coenzyme transport and metabolism (2.53%), and 177 CDSs were associated with lipid transport and metabolism (3.05%).

Various putative gene clusters that may be involved in the mechanism behind bacterial secondary metabolite biosynthesis were identified, including the abyssomicin biosynthetic gene cluster and the desferrioxamine B biosynthetic gene cluster.

### Data availability.

This whole-genome shotgun project has been deposited at DDBJ/EMBL/GenBank under the accession number RQIV00000000. The SRA accession number is PRJNA505761.
